# Feeling (in)complete: neural correlates of perceived body integrity in individuals with lower limb amputation

**DOI:** 10.1186/s12984-025-01817-3

**Published:** 2026-01-05

**Authors:** Robin Bekrater-Bodmann, Michaela Ruttorf

**Affiliations:** 1 Department of Psychiatry, Psychotherapy and Psychosomatics, Uniklinik RWTH Aachen, Aachen, Germany; 2 Scientific Center for Neuropathic Pain Aachen SCNAACHEN, Uniklinik RWTH Aachen, Roermonder Straße 110, 52072 Aachen, Germany; 3 Institute of Cognitive and Clinical Neuroscience, Central Institute of Mental Health, Medical Faculty Mannheim, Heidelberg University, Mannheim, Germany; 4 Computer Assisted Clinical Medicine, Medical Faculty Mannheim, Heidelberg University, Mannheim, Germany; 5 Mannheim Institute for Intelligent Systems in Medicine, Medical Faculty Mannheim, Heidelberg University, Mannheim, Germany

**Keywords:** Amputee, Body ownership, FMRI, Posterior parietal cortex, Rubber hand illusion, Superior parietal cortex

## Abstract

**Background:**

The amputation of a limb constitutes one of the most severe disruptions of body integrity. Nevertheless, many individuals with limb amputation report a restored sense of integrity when wearing a prosthesis. The rubber limb illusion (RLI) has been proposed as an experimental model to study such experiences. In this paradigm, correlated visuo-tactile stimulation of the residual limb and an artificial limb can induce amputated individuals to experience ownership of the latter one which is then perceived as a counterpart of the missing limb. However, due to methodical limitations in previous setups, the neural processes underlying alterations in the sense of body integrity remain insufficiently understood.

**Methods:**

In this cross-sectional study, we developed a novel RLI setup to systematically manipulate the sense of body integrity in a sample of *N* = 34 individuals with unilateral lower limb amputation. Participants completed six randomized trials across two experiments. In Experiment 1, we varied artificial limb appearance (intact vs. impaired) and visuo-tactile stimulation (synchronous vs. asynchronous) on the residual limb. In Experiment 2, we manipulated artificial limb appearance and induced the RLI on both the residual and the non-amputated limb. Neural activity was assessed using functional magnetic resonance imaging.

**Results:**

Synchronous visuo-tactile stimulation of the residual limb and an intact artificial counterpart induced artificial limb ownership and was associated with improvements in perceived body integrity. Neuroimaging revealed specific activation in the left superior parietal lobule associated with dynamic changes in the sense of body integrity. Neural activity underlying RLI processing did not significantly differ between the residual limb and the non-amputated limb.

**Conclusion:**

Appropriate multimodal sensory stimulation can strengthen the sense of body integrity in most individuals with lower limb amputation. This effect appears to be mediated by the brain’s capacity for sensory integration within the body representation network. These insights advance our understanding of prosthesis-related experiences and may inform the development of improved prosthetic devices that employ non-invasive somatosensory feedback, thereby promoting positive rehabilitative outcomes through enhanced prosthesis embodiment.

**Supplementary Information:**

The online version contains supplementary material available at 10.1186/s12984-025-01817-3.

## Introduction

The amputation of a limb represents a profound disruption to an individual’s body integrity, with some individuals experiencing a persistent sense of physical incompleteness [1]. However, many individuals report a restored sense of body integrity when wearing their prosthesis [2]. This adaptation in body experience is closely linked to the phenomenon of prosthesis embodiment, which entails perceiving the device not merely as a tool but as an integral part of the body, with subjective ownership of the prosthesis contributing to the connection to the self [3]. Given the potential rehabilitative implications of prosthesis embodiment for the sense of body integrity in individuals with limb amputation [4], researchers are increasingly interested in its neurobehavioral underpinnings.

For nearly three decades, the rubber limb illusion (RLI) paradigm has served as an experimental model for investigating the principles underlying embodiment experiences. In the original setup, developed for upper limbs in non-amputated individuals [5], a participant’s hidden hand and a visible rubber hand are synchronously stroked. This causes about 75% of participants to experience ownership over the artificial limb, i.e., to feel as if the rubber hand were their own hand [6–9]. Asynchronous visuo-tactile stimulation reduces or even abolishes this effect [5, 10]. Recent results demonstrated that the RLI relies on bottom-up multimodal sensory integration [11] rather than top-down effects such as suggestibility or expectations [12]. The RLI paradigm can be flexibly modified based on multisensory integration principles [13] and induces similar effects across body parts, including the lower limbs [14, 15].

Crucially, also individuals with limb amputation can be induced to perceive the RLI when their hidden residual limb is touched in synchrony with an intact artificial limb [16]. However, the induction rate is significantly lower compared to non-amputated individuals, and successful illusion induction requires perceptual conditions—such as referred sensations where touch applied to the residual limb is referred to the phantom limb in a somatotopic manner [16]—that are present only in about one out of six individuals with limb amputation [17]. Neuroimaging in these rare cases [18] demonstrated involvement of premotor and parietal cortices, paralleling networks described for non-amputated individuals experiencing the RLI [19] and indicating that core integration capabilities of body-related sensory input at least partly persist after limb amputation. However, given the methodological challenges, the neurobehavioral processes underlying embodiment experiences in individuals with limb amputation remain underexplored, as does the understanding of how these processes are comparable between residual and non-amputated body parts.

Furthermore, although the neural processes underlying embodiment have been relatively well characterized in non-amputated participants, insights into RLI-induced alterations in the sense of body integrity—particularly relevant for individuals with limb amputation—are still lacking. In other clinical contexts, the superior parietal lobule (SPL) has been identified as a key region implicated in alterations of perceived body integrity [20–23], suggesting its essential role in integrating multisensory inputs to generate a unified body percept. It is therefore hypothesized that the SPL also contributes to the sense of body integrity in individuals with limb amputation when embodiment in the RLI paradigm is successfully induced.

The present study thus aims to elucidate the neural correlates of the RLI in terms of perceived body integrity in a robust sample of individuals with lower limb amputation. We specifically focused on the involvement of the SPL. In two functional magnetic resonance imaging (fMRI) experiments, we systematically manipulated the perception of body integrity by applying synchronous or asynchronous tactile stimulation to both the hidden residual limb and an intact or impaired artificial counterpart (Experiment 1) and compared the perceptual and neural effects to those observed when the illusion was induced between the residual and the non-amputated limb (Experiment 2). Experiment 1 thus allows for the identification of brain areas associated with the sense of body integrity, while Experiment 2 focuses on potential differences of RLI processing as a function of target limb presence or absence.

## Materials and methods

### Participants

Participants were recruited using a previously established sample of individuals with unilateral major lower limb amputation (e.g., [2–4]), who (a) had an age between 18 and 80 years, (b) spoke German fluently, and (c) used a prosthesis on a regular basis. There were no other inclusion or exclusion criteria.

We included the data of *N* = 34 individuals with lower limb amputation (three exclusions from fMRI analysis due to excessive head movements, see below). Of the total sample, *n* = 27 identified as male (no non-binary or gender-diverse participants). The mean (*M*) age was 55.85 years (standard deviation, *SD*, of 9.33 years). Twenty-two participants were amputated on the left leg. Nineteen participants had a transfemoral amputation, while the others had a transtibial one. Most participants reported a traumatic amputation which dated back on average *M* = 32.33 years (*SD* = 13.70; one missing value). Detailed participant characteristics are given in Table S1 (see Additional file 1). The study was approved by the ethics review board of the Medical Faculty Mannheim, Heidelberg University (reference number: 2016-658 N-MA), and was carried out in accordance with the Declaration of Helsinki in its current form. Written informed consent was obtained from all individuals prior to study participation. The study was registered in the German Clinical Trials Register (DRKS00028538).

### FMRI paradigm

We implemented a newly developed RLI paradigm within the environment of an MRI scanner to probe participants’ sense of body integrity. For this purpose, we built upon recent findings highlighting two distinct principles of conscious body perception. First, synchronous visuo-tactile signals from a single body part facilitate the emergence of the sense of ownership for adjacent body parts [24]. In other words, if, for example, an artificial leg is visually touched during the RLI experiment, ownership extends to the visible but unstimulated artificial foot. Second, a visual connection between the artificial limb and the rest of the body is crucial for eliciting the illusion of ownership [25, 26]. This means ownership of the unstimulated artificial foot can only be induced if it is visually connected to the stimulated artificial leg. We combined these two principles to experimentally modulate the participant’s experience of an intact or impaired body.

**Fig. 1:**
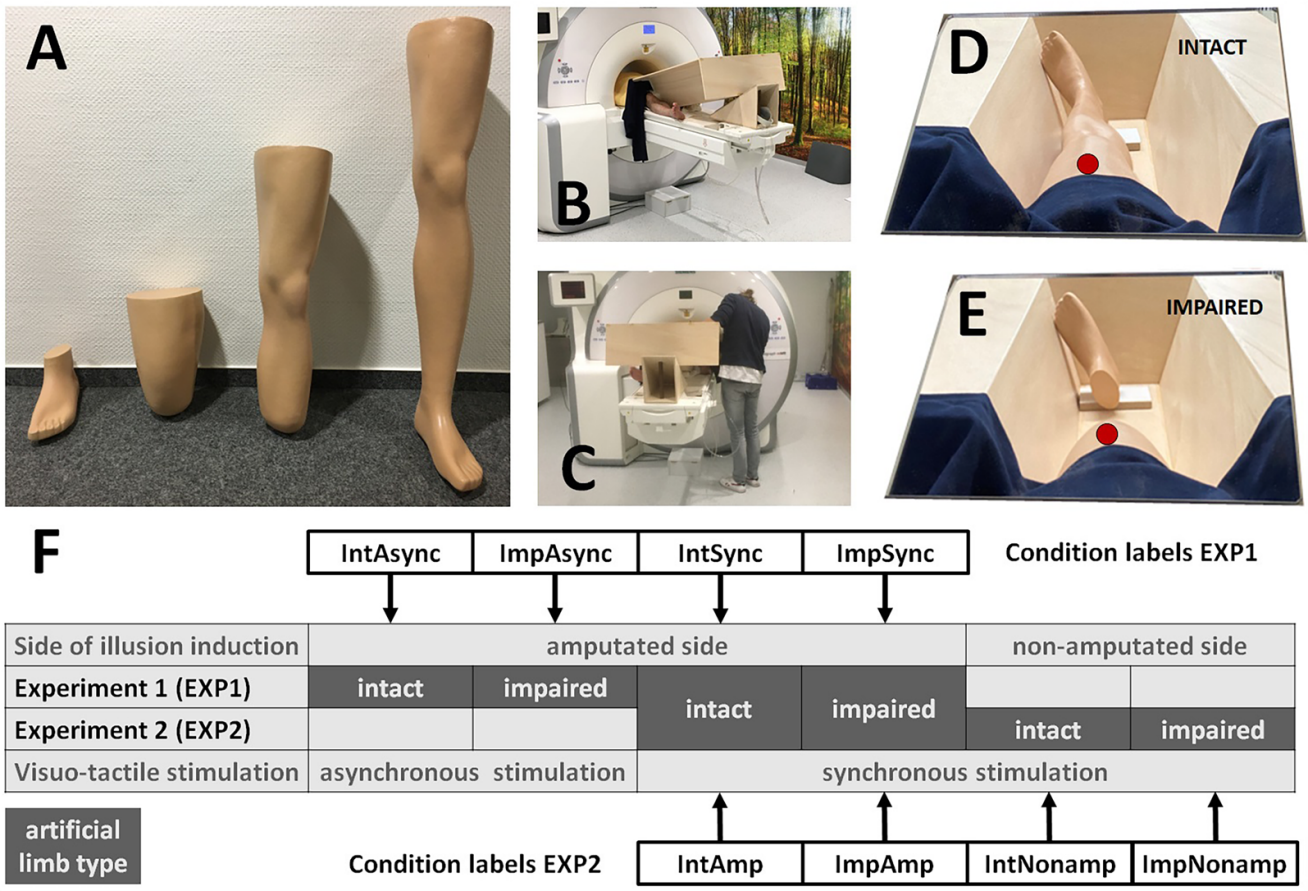
Rubber limb illusion setup. **A** The set of artificial limbs used in the present study, from left to right: intact limb, residual limb I (transtibial amputation), residual limb II (transfemoral amputation), foot; given are the artificial limbs for the left body side. **B** Participant’s position in the MRI scanner, with the wooden setup placed between both limbs. **C** Experimenter’s position during the MRI measurement. **D** Participant’s perspective during the measurement (intact-condition). **E** Participant’s perspective during the measurement (impaired-condition). The red markings indicate the area of tactile stimulation and match the respective stimulation area on the participant’s real limb. **F** Design of the study and allocation of trials to the two experiments. Condition labels are composed of two relevant factors out of the three possible components (side of illusion induction, artificial limb type, visuo-tactile stimulation), depending on which were manipulated in the given experiment. For example, *IntAsync* refers to an intact (= *Int*) artificial limb under asynchronous (= *Async*) visuo-tactile stimulation; since Experiment 1 was conducted only on the amputated side, side of illusion induction is not further specified in the condition label. And *ImpNonamp* refers to an impaired (= *Imp*) artificial limb with the illusion induced on the non-amputated (= *Nonamp*) side; since Experiment 2 only applied synchronous touches, visuo-tactile stimulation is not further specified in the condition label

To this aim, the Center for Orthopedic and Trauma Surgery (Heidelberg University Hospital, Heidelberg, Germany) modified artificial legs from a full-sized mannequin such that (a) left and right intact legs, (b) left and right residual limbs amputated at the transfemoral level, (c) left and right residual limbs amputated at the transtibial level, and (d) left and right feet were obtained (Fig. 1A). All artificial limbs were coated to resemble real prostheses by selecting the skin color most frequently chosen at the Center for Orthopedic and Trauma Surgery.

For the experiment, participants were placed in the MRI scanner. The head coil, equipped with a mirror, allowed participants to view their body along its length. The participant’s lower limbs were slightly spread apart, and a customized wooden construction was placed in the resulting gap. This construction prevented participants from seeing their own lower limbs while providing the experimenter sufficient space to apply touches during the MRI measurement (Fig. 1B, C). Additionally, the wooden construction created a space between the participants’ lower limbs into which an artificial limb could be positioned.

In separate trials, we used either an intact artificial lower limb (intact-conditions) or an impaired artificial lower limb, i.e., an artificial residual limb (impaired-conditions), adapted to a participant’s level of amputation (transfemoral or transtibial amputation, respectively). Crucially, in the impaired-conditions, an unconnected foot was positioned to match the foot location in the intact-conditions (see Fig. 1D, E). Fabric was applied on the side of the construction facing the participant to simulate a natural transition between the body and the artificial leg. Based on the feedback of the participants, the wooden construction was tilted (between 10° and 20°) until he or she had a full view of the artificial limb.

Together with the participant, the experimenter then specified the area of stimulation on the real lower limbs as well as the area of perceived correspondence on the artificial lower limbs. The area of stimulation was determined based on the real residual limb: the experimenter selected an area that was as distal as possible, while avoiding scarred regions, numb areas, or zones eliciting referred sensations to the phantom limb. On the real intact limb, an analogue area was defined. The experimenter marked the areas (distal-to-proximal length: approximately 5 cm) on both real limbs and transferred these marks to all artificial limbs used for that participant (see the markings in Fig. 1D, E).

A trained experimenter remained in the MRI scanner room during data acquisition and manually applied tactile stimuli to both the artificial limb and the real target lower limb using brushes. Paced by a jittered auditory signal, the experimenter applied the tactile stimuli either synchronously or asynchronously, with a mean frequency of about 0.5 Hz. Participants were instructed to observe the artificial limb and focus their attention on both the artificial leg (that is, the stimulated part of the fake limb) and the artificial but unstimulated foot, as they were asked for perceptions of both body parts afterwards. One trial lasted about 5 min and contained five stimulation blocks with a duration of 26.9 s each (ON blocks), alternating with six blocks without stimulation (OFF blocks) with the same duration. During OFF blocks, the experimenter’s hand was held still in the view of the participant who was asked to further observe the artificial body parts (as in the ON blocks).

We implemented six separate conditions (that is, six trials, one condition per trial) in randomized order: four conditions were implemented with the real residual limb as the stimulation target, where the type of artificial limb and the type of stimulation were systematically manipulated in a 2 (artificial limb; intact vs. impaired) ×2 (visuo-tactile stimulation; synchronous vs. asynchronous) design (Experiment 1). The resulting conditions were named as.

- *IntAsync* (intact-condition with asynchronous visuo-tactile stimulation),
- *ImpAsync* (impaired-condition with asynchronous visuo-tactile stimulation),
- *IntSync* (intact-condition with synchronous visuo-tactile stimulation), and.
- *ImpSync* (impaired-condition with synchronous visuo-tactile stimulation).

In this design, the intact vs. impaired manipulation controls for visual appearance effects of the artificial limb, the synchronous vs. asynchronous manipulation controls for the necessity of visuo‑tactile congruency, and their interaction tests whether the impact of visuo-tactile congruency on embodiment depends on the visual appearance of the artificial limb.

Two additional conditions (synchronous stimulation applied to the real non-amputated lower limb as target and an intact or an impaired artificial limb) were used to implement another 2 (body side; amputated vs. non-amputated) ×2 (artificial limb; intact vs. impaired) design (Experiment 2). The resulting conditions were named as.

- *IntAmp* (intact-condition on the residual limb),
- *ImpAmp* (impaired-condition on the residual limb),
- *IntNonamp* (intact-condition on the non-amputated limb), and.
- *ImpNonamp* (impaired-condition on the non-amputated limb).

Note that condition *IntSync* (Experiment 1) is identical to condition *IntAmp* (Experiment 2), and condition *ImpSync* (Experiment 1) is identical to condition *ImpAmp* (Experiment 2). In this design, the residual limb vs. non‑amputated limb manipulation controls for the influence of body side, the intact vs. impaired manipulation controls for visual appearance effects of the artificial limb, and their interaction tests whether the impact of visual limb appearance on embodiment differs depending on whether stimulation is applied to the amputated or non‑amputated body side. The conditions and their allocation to the two experiments are visualized in Fig. 1F. Between conditions, participants were instructed to close their eyes to prevent them from observing the experimenter’s adjustments to the setup for the next trial.

After each condition, participants were asked to rate statements about ownership experiences and perceived body integrity during the trial that were verbally communicated to them. Crucially, participants were given the identical set of ownership items, both for the leg (that is, the stimulated part of the artificial limb) and the foot (that is, the connected or disconnected unstimulated part of the artificial limb). To better evaluate these ratings, participants were additionally asked to report their perception of touch referral from the real to the artificial limb (the results of which are only reported in Additional file 1). The eight items (Table 1), presented in randomized order, were adapted from a recently validated questionnaire for the assessment of prosthesis embodiment in individuals with lower limb amputation [2], and were assigned a 7-point Likert scale ranging from 0 ‘strongly disagree’ to 6 ‘strongly agree’. The mean values of the items belonging to a given dimension (that is, leg ownership, foot ownership, body integrity, and referral of touch) were used in the subsequent analyses.

**Table 1:** Perceptions assessed during the experiment

Measure	Item wording
Leg ownership	I felt as if the artificial **leg** was part of my body
	It seemed as if the artificial **leg** belonged to me
Foot ownership	I felt as if the artificial **foot** was part of my body
	It seemed as if the artificial **foot** belonged to me
Body integrity	I felt physically complete
	I had the feeling of having two intact legs
Referral of touch	I felt the touch in the artificial leg
	The touch I observed felt like a real touch

Note that the items of both ownership categories were identically worded and differed only with respect to the target body part (leg vs. foot; highlighted in **bold**)

### Data acquisition and pre-processing

#### MRI measurements

All MRI data were acquired on a 3 T MAGNETOM Biograph whole body MRI scanner (Siemens Healthineers, Erlangen, Germany) using a 32-channel head coil. Structural MRI data were acquired using a 3D T_1_-weighted magnetization prepared rapid gradient echo (MPRAGE) sequence (repetition time (TR) = 1900 ms, echo time (TE) = 2.49 ms, flip angle (α) = 9°, field of view (FoV) = 240 × 240 mm^2^, matrix size = 256 × 256, bandwidth (BW) = 180 Hz/px, parallel acquisition technique GRAPPA acceleration factor 2) recording 192 sagittal slices. For acquisition of functional MRI (fMRI) data, a T_2_*-weighted gradient echo echo planar imaging (EPI) sequence (TR = 3190 ms, TE = 33 ms, FoV = 220 × 220 mm^2^, matrix size = 96 × 96, α = 90°, BW = 1270 Hz/px) with GRAPPA acceleration factor 3 was used. Each image volume consisted of 50 contiguous axial slices (slice thickness = 2.3 mm, gap = 0.7 mm) recorded in descending slice order oriented parallel to the line connecting the anterior commissure to the posterior commissure covering the whole brain. Before pre-processing, the first three volumes were discarded to allow for T_1_ saturation effects.

#### FMRI processing

Pre-processing, single subject, and group analyses were conducted with Statistical Parametric Mapping software (SPM12 v7771, Wellcome Trust Centre for Neuroimaging, Institute of Neurology, University College London, UK) implemented on MATLAB R2013a. Functional volumes were slice time corrected to reference slice #25 (middle slice in time) and realigned to the fourth volume by minimizing the mean square error (rigid body transformation) in order to correct for head movement. Three participants were excluded because of excessive motion estimates (greater than 2 mm in translation and 2° in rotation in at least one trial), resulting in a final sample of *N* = 31 participants for fMRI analyses. The created mean image was coregistered to each participant’s MPRAGE which was normalized into standard stereotactic space (Montreal Neurological Institute, Quebec, Canada) using tissue probability maps in SPM12. The nonlinear transformation parameters were then applied to the functional images. To reduce spatial noise, the images were smoothed with a 6.9 × 6.9 × 9.0 mm^3^ full-width at half maximum Gaussian kernel. To remove low-frequency noise, a high-pass filter (cutoff 1/128 Hz) was included, and the time series were corrected for serial autocorrelations using first-order autoregressive models (AR(1)). For each of the six trials, a fixed effects analysis was performed by setting up a general linear model (GLM) including the following experimental conditions: visuo-tactile stimulation on (ON) and visuo-tactile stimulation off (OFF). These inputs were convolved with a canonical hemodynamic response function (first-order expansion) to create the design matrix. The six parameters describing the rigid body transformation were implemented as confound variables in the statistical analyses to covary out signals correlated with head motion. On a second-level analysis, individual contrasts ON minus OFF were employed using a within-subject ANOVA design with four sessions per participant per experiment. Results were considered significant at *P* < 0.05 on a voxel-level, either whole-brain or small-volume corrected for multiple comparisons using a family-wise error (FWE) or false discovery rate (FDR). For small-volume correction, regions of interest (ROIs) were defined by means of SPM Anatomy Toolbox (Version 3.0) [27]. A joint ROI was created from all areas (i.e., 5Ci, 5 L, 5 M, 7 A, 7 M, 7P, 7PC) corresponding to left and right superior parietal lobule (SPL), respectively, and saved to file. Neural activity (in terms of beta values) of significant clusters was extracted from a spherical ROI with a 10 mm radius centered on the peak coordinate. The ROI’s beta values were extracted from each participant’s contrast image and used for further statistical analyses. For this purpose, a one-sample *t* test with individual contrasts ON minus OFF was computed for each of the sessions separately. For extraction of beta values, MarsBaR [28] was used.

Based on previous results on the RLI (e.g., [4–6]), we further (a) created a ROI representing the bilateral intraparietal sulcus using the Anatomy toolbox (i.e., hlP1, hlP2, hlP3) and (b) used a previously introduced ROI representing the bilateral ventral premotor cortex (PMv) [29, 30]. Since the whole-brain results suggest an extended brain network responding to the RLI setup in the present study (see below), we also created bilateral ROIs (using the Anatomy toolbox) for the secondary somatosensory cortex and its vicinity (SII+; OP1, OP2, OP3, OP4) and the middle temporal complex (MT+; hOc5 [V5/MT]) to evaluate specificity of its involvement.

#### Statistics for perceptual data

Descriptive analyses of all included variables were performed, and prevalence (%), *M*, and *SD* are provided, based on the scaling level. Mean values for the different dimensions of embodiment (i.e., leg ownership, foot ownership, and body integrity; referral of touch serves as manipulation check and is reported only in Additional file 1) in the experiment were calculated and are provided together with their standard deviation.

A mean rating of at least 3/6 was used to classify participants as responders for a given perceptual measure, as values above the midpoint reflect a shift towards agreement, rather than disagreement, regarding the emergence of perception. We further identified “overall responders” (*n* = 18 of the entire sample, *n* = 16 for those with valid neuroimaging data), characterized by showing these ratings across all perceptual measures in the crucial *IntSync* condition. The other participants were considered low or non-responders (*n* = 16 of the entire sample, *n* = 15 for those with valid neuroimaging data). The rationale behind this approach was to ensure comparability with previous research (e.g., [6, 7, 16]) that included only previously identified RLI responders. Accordingly, our design provides both unbiased results from the entire sample and targeted insights into the neural processes underlying body perception within the subsample of overall responders. In doing so, our approach contributes to a more comprehensive understanding of the underexplored principles governing the multisensory-mediated sense of body integrity in individuals with limb amputation.

To accommodate violations of statistical assumptions, we rank-transformed our perceptual data and used rank-based analyses of variance (ANOVAs) that provide robust results and further allow for an interpretable evaluation of interactions in our design [31]. Perceptual data were entered in separate rank-based 2 × 2 repeated measurements ANOVAs: a 2 (artificial limb; intact vs. impaired) ×2 (visuo-tactile stimulation; synchronous vs. asynchronous) design (Experiment 1), and a 2 (body side; amputated vs. non-amputated) ×2 (artificial limb; intact vs. impaired) design (Experiment 2). For each ANOVA, *F*-values, *P*-values, and *η*^2^ as effect size measure are reported. Whenever an interaction became significant, simple effect analysis was performed using paired *t*-tests with Bonferroni-corrected *P*-values (*P*_Bonf_) and Cohen’s *d* as effect size measure. If not indicated otherwise, statistical analyses were conducted using two-tailed testing. We employ a hierarchical interpretation of ANOVA results to ensure correct inference when both main effects and interactions were tested. Specifically, if a statistically significant interaction is present, interpretation focuses on the interaction term, as it indicates that the effect of one factor depends on the level of another. Main effects are always reported but only interpreted if the interaction is not statistically significant, reflecting the logic that higher-order effects take precedence in the explanation of variance in factorial designs. Extracted neural data (non-transformed) were analysed accordingly. Pearson correlations were performed whenever relationships between two variables were evaluated. All statistical analyses were performed using MS Excel and IBM SPSS v29.

## Results

### Experiment 1: rubber limb illusion induction on the residual limb

#### Perceptual data

##### Leg ownership

**Fig. 2:**
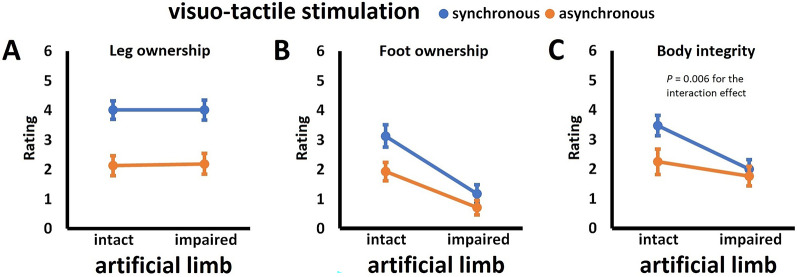
Perceptual results for Experiment 1. Given are the mean ratings +/- standard errors of the mean (higher values indicate higher agreement to items asking for the given measure) for (**A**) leg ownership, (**B**) foot ownership, and (**C**) body integrity. Displayed are the non-transformed data of *N* = 34 participants; statistical details are provided in the main text

The ANOVA revealed no significant main effect for the factor *artificial limb*, *F*(1,33) = 0.01, *P* = 0.91, *η*^2^ < 0.001, but there was a significant main effect for the factor *visuo-tactile stimulation*, *F*(1,33) = 53.14, *P* < 0.001, *η*^2^ = 0.62, with synchronous stimulation being associated with significantly higher ratings of leg ownership than asynchronous stimulation. There was no significant *artificial limb × visuo-tactile stimulation* interaction, *F*(1,33) = 0.02, *P* = 0.89, *η*^2^ = 0.001. These results suggest that synchronous visuo-tactile stimulation induces comparable ownership over an artificial leg, independent of whether it is intact or impaired, which is visualized in Fig. 2A. Accordingly, responder rate in this measure was high for synchronous (*IntSync*: 79.4%; *ImpSync*: 82.4%) compared to asynchronous conditions (*IntAsync*: 35.3%; *ImpAsync*: 35.3%). The pattern of touch referral observed in Experiment 1 parallels the pattern of leg ownership (for details, see the analysis of touch referral data in Additional file 1, Suppl. Fig. S1A).

##### Foot ownership

There was a significant main effect for the factor *artificial limb*, *F*(1,33) = 35.77, *P* < 0.001, *η*^2^ = 0.52, with an intact limb being associated with significantly higher foot ownership ratings than an impaired one. There was also a significant main effect for the factor *visuo-tactile stimulation*, *F*(1,33) = 13.46, *P* < 0.001, *η*^2^ = 0.29, with synchronous stimulation being associated with significantly higher ratings of foot ownership than asynchronous stimulation. There was no significant *artificial limb* × *visuo-tactile stimulation* interaction, *F*(1,33) = 2.59, *P* = 0.12, *η*^2^ = 0.07. These results suggest that both synchronous visuo-tactile stimulation and artificial limb intactness independently contribute to perceived ownership over an unstimulated artificial foot. These results are visualized in Fig. 2B. Only in the *IntSync* condition, most participants (i.e., 55.9%) were identified as responders in this measure (*ImpSync*: 20.6%; *IntAsync*: 32.4%; *ImpAsync*: 11.8%).

##### Body integrity

The analysis of the body integrity measure revealed a significant main effect for the factor *artificial limb*, *F*(1,33) = 16.30, *P* < 0.001, *η*^2^ = 0.33, with an intact artificial limb being associated with significantly higher body integrity ratings than an impaired one. There also was a significant main effect for the factor *visuo-tactile stimulation*, *F*(1,33) = 11.80, *P* = 0.002, *η*^2^ = 0.26, with synchronous stimulation being associated with significantly higher ratings of body integrity than asynchronous stimulation. Crucially, there was also a significant *artificial limb* × *visuo-tactile stimulation* interaction, *F*(1,33) = 8.45, *P* = 0.006, *η*^2^ = 0.20. Simple effects analyses revealed that the interaction was driven by significantly higher ratings in the *IntSync* condition compared to all other conditions, all *T*(33) ≥ 4.09, all *P*_*Bonf*_ < 0.001, all *d* ≥ 0.70. Only in the *IntSync* condition, the majority of participants (70.6%) was identified as responders in this measure (*ImpSync*: 38.2%; *IntAsync*: 41.2%; *ImpAsync*: 29.4%). These results are visualized in Fig. 2C.

##### Correlations between perceptual measures

In the crucial *IntSync* condition, the measures for leg ownership, foot ownership, and body integrity were highly correlated to each other, all *r*(33) between 0.67 and 0.83, all *P*_*Bonf*_ < 0.001, suggesting at least partly shared perceptual processes underlying the phenomena. Most participants (i.e., 52.9%) were classified as overall responders.

#### FMRI results

**Fig. 3:**
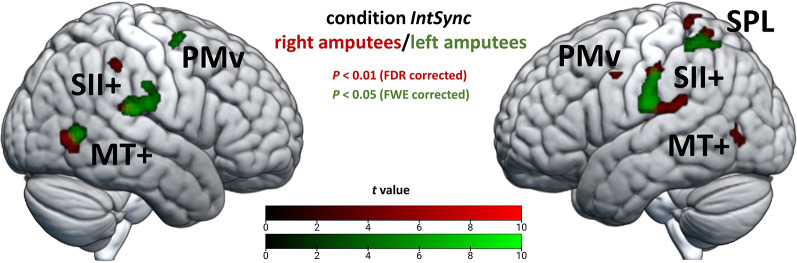
Activation patterns for right and left amputees for condition ***IntSync***. The activation patterns of *N* = 31 individuals with right (red) and left (green) lower limb amputation for condition *IntSync* are shown (for illustrative purposes only). Both right and left amputees show activation in left superior parietal lobe (SPL), but not in right SPL. The different significance levels were chosen to compensate for the varying sample sizes. The cluster extension threshold is *k* = 10. PMv = ventral premotor cortex; SII + = secondary somatosensory cortex and its vicinity; MT + = middle temporal complex

For the *IntSync* condition, separate whole-brain analyses of the two groups (individuals with right and left amputation) provided similar results regarding the significant clusters. Particularly, there was correspondence regarding the involvement of bilateral SII + and MT+. Crucially, in both groups, there was stronger activity in the left-sided posterior parietal cortex, suggesting functional lateralization (Fig. 3). Therefore, we merged the two groups for further analyses. Table 2 provides the brain activation in the merged group for the *IntSync* condition, now revealing significant activation in a network comprising bilateral SII+, MT+, PMv, and posterior parietal cortex. To a varying degree, this network was involved in the other conditions of Experiment 1 as well (Table S2-S4, see Additional file 1). Crucially, however, only in the *IntSync* condition, peak activity within the bilateral posterior parietal cortex cluster shifted to the superior parietal lobule (SPL).

**Table 2:** Results for whole-brain analysis-contrast *IntSync*/*IntAmp*

Functional unit	Atlas label (AAL)	Hemi-sphere	MNI coordinates			Peak t-value	Voxels per cluster
			x	y	z		
	Parietal Inf	L	−42	−45	56	7.96	220
		L	−39	−57	56	7.44	
SPL	Parietal Sup	L	−35	−52	63	8.49	
		L	−25	−61	63	7.09	
		R	41	−57	54	6.49	27
		R	41	−50	58	5.59	
	Postcentral	R	55	−20	35	7.28	319
SII+	Supra Marginal	R	60	−20	24	7.78	
	Rolandic Oper	R	55	−29	19	9.98	
	Parietal Inf	L	−55	−22	40	8.98	297
		L	−58	−27	49	6.47	
	Postcentral	L	−58	−20	19	8.63	
	Temporal Sup	L	−53	−32	17	6.19	
MT+	Temporal Mid	R	46	−61	3	8.85	171
		L	−51	−71	1	7.07	127
		L	−42	−66	5	6.85	
PMv	Precentral	R	55	8	42	7.01	34
		L	−53	3	42	6.34	22
	Frontal Mid 2	R	39	−2	60	7.05	32

All peak t-values listed are significant after whole-brain correction for multiple comparisons using a family-wise error rate (*P* < 0.05, FWE-corrected at voxel level) with extent threshold *k* > 20 voxels. For clusters *k* > 100, all peak values are denoted. Functional unit refers to the brain regions (MT+, medial temporal complex; PMv, ventral premotor cortex; SII+, secondary somatosensory cortex and its vicinity, including Rolandic operculum, supramarginal cortex, and adjacent parts of the inferior parietal lobule) that were consistently identified, as well as to the superior parietal lobule (SPL), and is intended to facilitate the assignment to description in the results section. L/R denotes the left and right hemisphere, respectively. Atlas labels according to Automatic Anatomical Labelling (AAL) Atlas 3. MNI, Montreal Neurological Institute

**Fig. 4:**
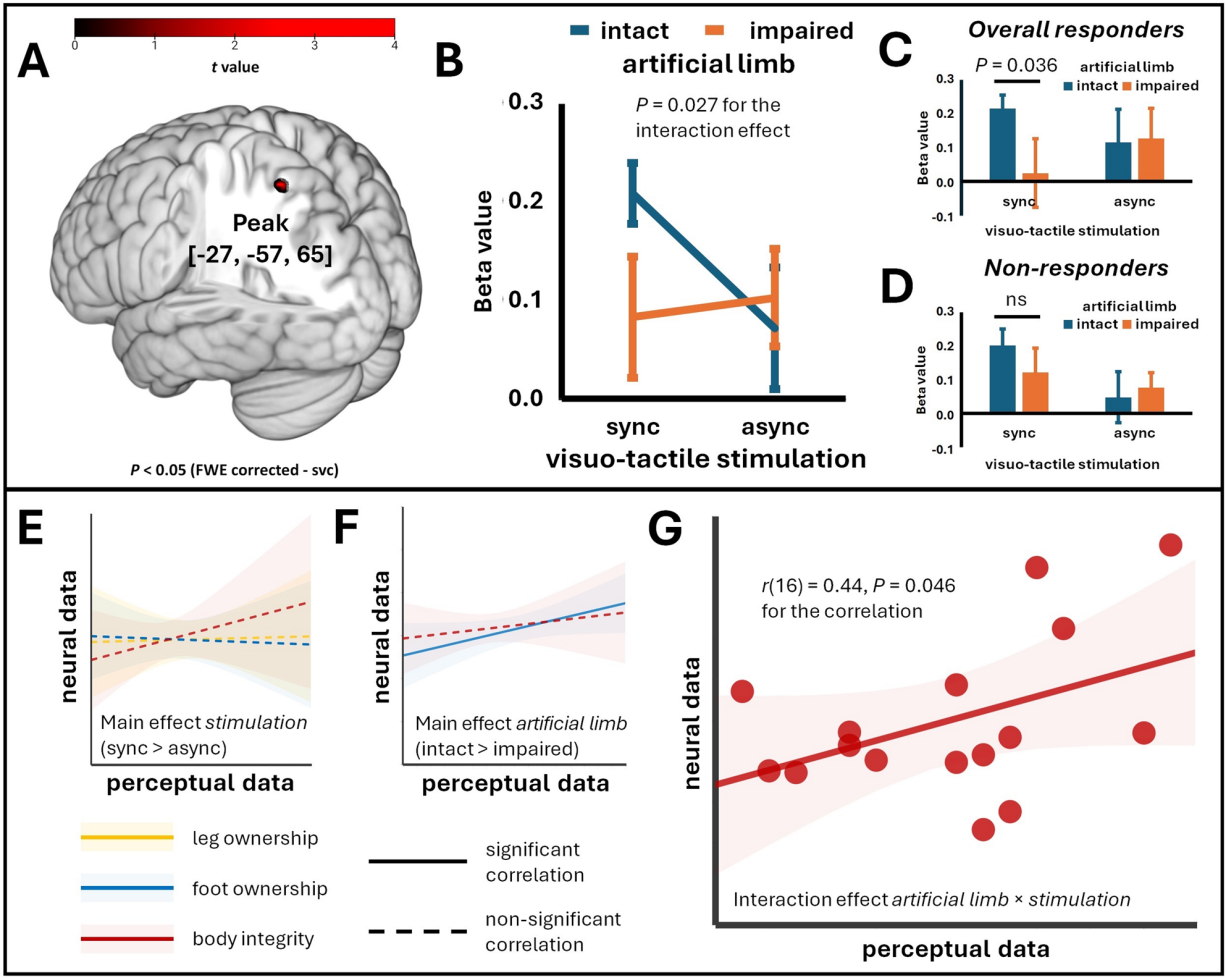
Superior parietal lobule (SPL) activation in Experiment 1. **A** The activation pattern for the interaction term (*IntSync + ImpAsync*)—(*IntAsync + ImpSync*) for *N* = 31 participants is shown. The results are small-volume corrected (svc) for multiple comparisons using a family-wise error rate (FWE, *P* < 0.05, voxel-level and cluster-level). For small-volume correction, atlas masks of left and right superior parietal lobe were used, the minimum number of statistically significant voxels per cluster (FWEc) is *k* = 5. **B** Interaction diagram for extracted beta values from the left SPL (extracted from a spherical region of interest with a 10 mm radius centered on the peak coordinate [−25, −57, 65]) for *N* = 31; sync = synchronous; async = asynchronous. **C** Extracted neural data (left SPL) for *n* = 16 overall responders. **D** Extracted neural data (left SPL) for *n* = 15 non-responders. Note that a *t* test revealed significantly different SPL responses to synchronous visuo-tactile stimulation as a function of the artificial limb used in overall responders, but not in non-responders (one-tailed; ns = not significant). Given are the mean ratings +/- standard errors of the mean. **E** Regression lines and 95% confidence intervals for the relationship between the perceptual and neural main effects of the factor *visuo-tactile stimulation* (synchronous > asynchronous) for the perceptual measures (*n* = 16 overall responders). **F** Regression lines and 95% confidence intervals for the relationship between the perceptual and neural main effects of the factor *artificial limb type* (intact > impaired) for the perceptual measures (*n* = 16 overall responders). **G** Scatterplot with regression line and 95% confidence interval for the correlation between the interaction effects for neural activity and perceived body integrity (*n* = 16 overall responders). Units in the plots were omitted due to their arbitrary nature. Displayed are the non-transformed data

Analysis of body integrity data (see above) suggested a significant interaction between the factors *artificial limb* and *visuo-tactile stimulation*, with analysis of simple effects revealing that an improved body integrity percept can be induced by observing an intact artificial limb that is touched in synchrony with the hidden residual limb. In accordance with these analyses, we generated the corresponding interaction term for fMRI data. In this analysis, no active voxel survived whole-brain FWE correction. By applying small volume correction using the bilateral SPL ROI, we found significantly active voxels only in left (peak activity in [−25, −57, 65]), but not in right SPL (Fig. 4A). Remarkably, the pattern of interaction in the neural data (Fig. 4B) parallels the pattern of interaction for body integrity (Fig. 2C). Subsequent analyses comparing overall responders and non-responders indicated that the former drive the interaction through a particularly dynamic response of their left SPL to synchronous visuo-tactile stimulation of their real residual limb together with an intact artificial limb (Fig. 4C, D). The interaction was specific for the left SPL, since we did not find it for the ROIs representing PMv, intraparietal sulcus, SII+, or MT+.

In the next step, we investigated the relationship between neural activity and perceptual effects in the group of overall responders. Specifically, we used the extracted beta values of neural activity from the left SPL and correlated their main or interaction effects at the neural level with the corresponding perceptual effects that were found to be significant (note that the correlation for main effects in the body integrity measure cannot be meaningfully interpreted due to the significant interaction effect). The main effects of *visuo-tactile stimulation* on perceptual measures did not significantly correlate with neural activity (*r*(16) = −0.03 for leg ownership, *r*(16) = −0.05 for foot ownership; both *P* (one-tailed) ≥ 0.43, Fig. 4E), indicating that somatosensory stimulation alone does not covary with activity in left SPL. A significant positive correlation was observed between the neural main effect of the *artificial limb* factor and the corresponding perceptual effect on foot ownership (*r*(16) = 0.48, *P* (one-tailed) = 0.030, Fig. 4F), suggesting that the left SPL exhibits perception-related differential responses to visual input from an intact limb. Crucially, for body integrity, the neural interaction effect was positively correlated with the corresponding perceptual effect, *r*(16) = 0.44, *P* (one-tailed) = 0.046 (Fig. 4G).

### Experiment 2: rubber limb illusion on the residual limb compared to the non-amputated limb

#### Perceptual data

##### Leg ownership

**Fig. 5:**
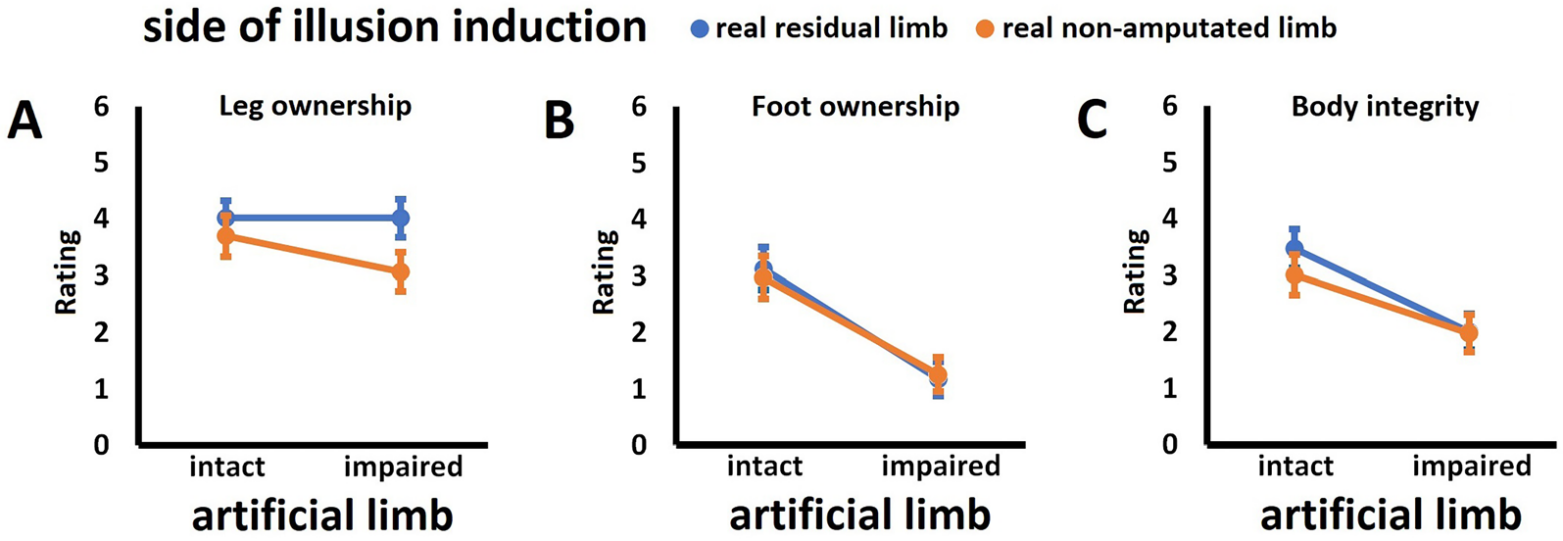
Perceptual results for Experiment 2. Given are the mean ratings +/- standard errors of the mean (higher values indicate higher agreement to items asking for the given measure) for (**A**) leg ownership, (**B**) foot ownership, and (**C**) body integrity. Displayed are the non-transformed data of *N* = 34 participants; statistical details are provided in the main text

The ANOVA revealed no significant main effect for the factor *artificial limb*, *F*(1,33) = 1.96, *P* = 0.17, *η*^2^ = 0.06. However, there was a significant main effect of the factor *body side*, *F*(1,33) = 15.60, *P* < 0.001, *η*^2^ = 0.32, with illusion induction on the real residual limb being associated with significantly higher ratings than illusion induction on the real non-amputated limb. There was no significant *artificial limb × body side* interaction, *F*(1,33) = 2.63, *P* = 0.11, *η*^2^ = 0.07. These results suggest that participants are more prone to perceive illusory leg ownership when synchronous visuo-tactile stimulation is applied to the real residual limb and an artificial limb (Fig. 5A). Crucially, the responder rate in the *IntNonamp*, *IntAmp*, and *ImpAmp* conditions was similar (73.5%, 79.4% and 82.4%, respectively), and slightly lower in the *ImpNonamp* condition (61.8%). The pattern of touch referral data in Experiment 1 parallels the pattern of leg ownership data (for details, see the analysis of touch referral data in Additional file 1, Suppl. Fig. S1B).

##### Foot ownership

There was a significant main effect for the factor *artificial limb*, *F*(1,33) = 34.25, *P* < 0.001, *η*^2^ = 0.51, with an intact limb being associated with significantly higher foot ownership ratings than an impaired limb. There was neither a significant main effect for the factor *body side*, *F*(1,33) = 0.01, *P* = 0.91, *η*^2^ < 0.001, nor a significant *artificial limb* × *body side* interaction, *F*(1,33) = 0.85, *P* = 0.36, *η*^2^ = 0.02, suggesting that the intactness of the artificial limb is equally important for the induction of foot ownership on both the amputated and the non-amputated body side (Fig. 5B). Most participants were classified as responders in the intact-conditions (61.8% for the *IntNonamp* condition and 55.9% for the *IntAmp* condition), whereas this was not the case in the impaired-conditions (20.6% for both the *ImpNonamp* and *ImpAmp* conditions).

##### Body integrity

The analysis of the body integrity measure revealed a significant main effect for the factor *artificial limb*, *F*(1,33) = 22.72, *P* < 0.001, *η*^2^ = 0.41, but no significant main effect for the factor *body side*, *F*(1,33) = 1.79, *P* = 0.19, *η*^2^ = 0.05, and no significant *artificial limb × body side* interaction, *F*(1,33) = 1.83, *P* = 0.19, *η*^2^ = 0.05. Most participants were classified as responders in the intact-conditions (61.8% for the *IntNonamp* condition and 70.6% for the *IntAmp* condition), but not in the impaired-conditions (38.2% for both the *ImpNonamp* and *ImpAmp* conditions). These results are visualized in Fig. 5C.

#### FMRI results

**Fig. 6:**
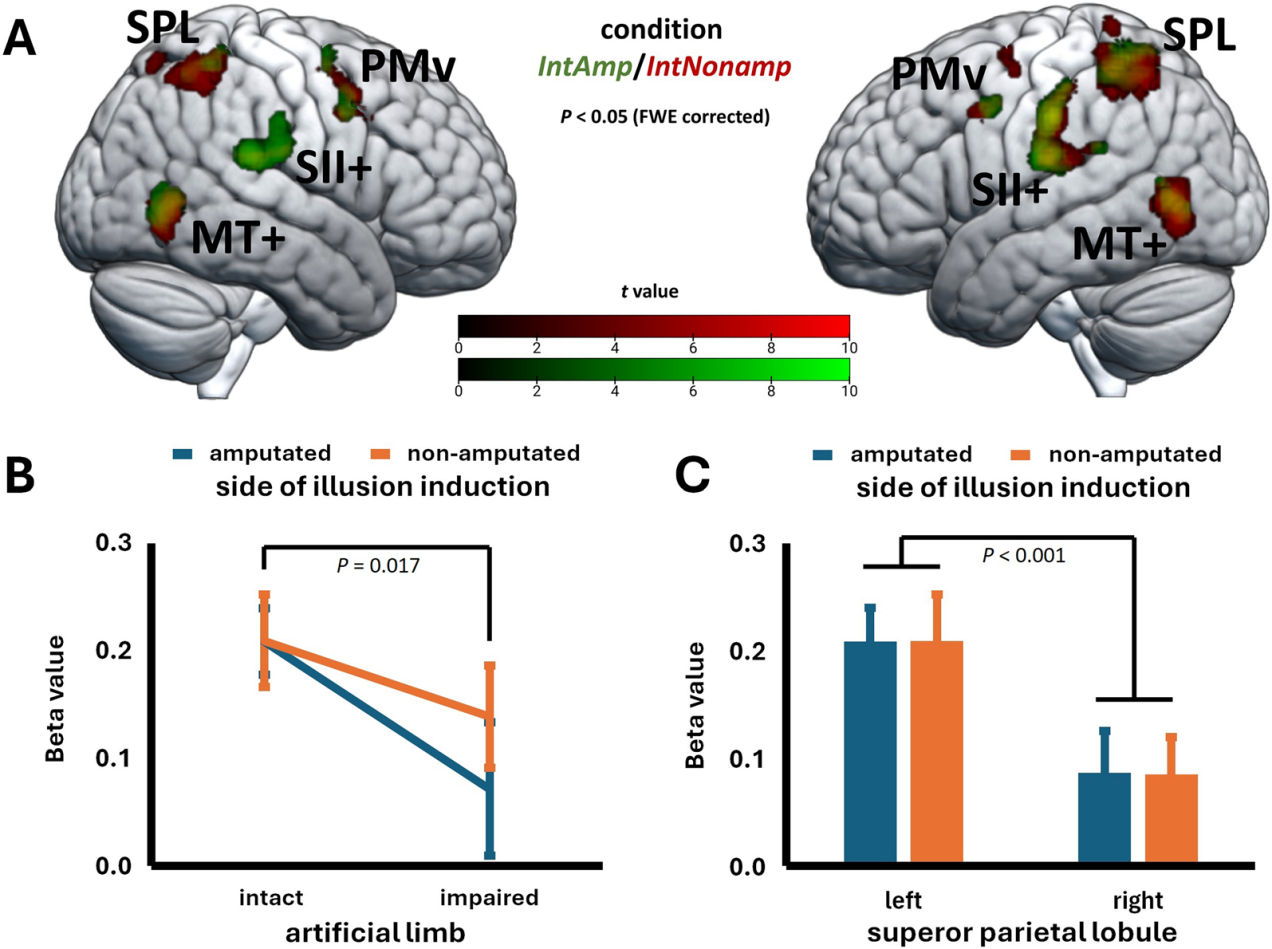
Superior parietal lobule (SPL) activation in Experiment 2. **A** The activation patterns for condition *IntAmp* (red) and condition *IntNonamp* (green) are shown. In both conditions, activation patterns are similar as expressed by the color-shifts (orange/yellow). The results are whole-brain corrected for multiple comparisons using a family-wise error rate (FWE, *P* < 0.05, voxel-level) and a cluster extension threshold of *k* = 10. PMv = ventral premotor cortex; SII + = secondary somatosensory cortex and its vicinity; MT + = middle temporal complex. **B** Diagram for extracted beta values in the left SPL (extracted from a spherical region of interest with a 10 mm radius centered on the peak coordinate as identified in Experiment 1 [−25, −57, 65]; *N* = 31). Given are the mean ratings +/- standard errors of the mean. **C** Bar graph for extracted beta values in the left SPL (extracted from a spherical region of interest with a 10 mm radius centered on the peak coordinate as identified in Experiment 1 [−25, −57, 65]) and the corresponding contralateral region (spherical region of interest with a 10 mm radius centered on [25, −57, 65]) of *N* = 31 participants (intact-conditions). Given are the mean ratings + standard errors of the mean

For conditions *IntAmp* and *IntNonamp*, whole-brain analyses provided similar results with regard to significant clusters. There was significant correspondence regarding bilateral SII+, MT+, and PMv activity (Table S5, S6, see Additional file 1; Fig. 6A). In both intact-conditions, we also found stronger bilateral SPL activity compared to the impaired-conditions. Notably, the pattern of effects (significant main effect for the factor *artificial limb*, *F*(1,30) = 6.36, *P* = 0.017, *η*^2^ = 0.17; no other main or interaction effects, all *P* ≥ 0.36, Fig. 6B) descriptively parallels the pattern for body integrity (Fig. 5C). However, no active voxels survived whole-brain FWE correction for the main effects of the factors *body side*, *artificial limb*, or its interaction, and even after applying small volume correction, no significant differences were detected in left SPL. When we compared the extracted beta values for the left and right SPL during Experiment 2’s intact-conditions, we observed a strikingly similar pattern (Fig. 6C): regardless of whether the illusion was induced on the residual or non-amputated limb, activity in the left SPL was strong, whereas activity in the right SPL was significantly weaker. The main effect of *hemisphere* (right vs. left SPL) was robust, *F*(1,30) = 23.23, *P* < 0.001, *η*^2^ = 0.44; however, there was neither a significant main effect of *body side* nor an interaction of the two factors (all *P* ≥ 0.98). This suggests that neural processes in the bilateral SPL associated with RLI induction are comparable for both the amputated and non-amputated limb. Neither whole-brain analysis nor small-volume analyses of PMv, SII+, and MT + revealed significant effects in these regions in Experiment 2.

## Discussion

In this study, we introduced a novel RLI paradigm to manipulate the sense of body integrity in individuals with lower limb amputation and examined its perceptual and neural correlates via fMRI. Synchronous visuo-tactile stimulation applied to the residual limb and an intact artificial limb induced illusory limb ownership and altered participants’ perception of the integrity of their body. Alterations in the sense of body integrity were accompanied by increased activity in the left superior parietal lobule (SPL), regardless of the side of amputation. Both perceptual experiences and neural responses were similar when stimulating either the residual or the non-amputated limb. Taken together, these findings suggest that the left SPL is specifically involved in processing information related to an individual’s body integrity when such information is presented visually or within a multisensory context including visual input, but that it is not activated by tactile input in isolation. The results therefore indicate that, despite bodily impairment, individuals with lower limb amputation can develop an improved sense of body integrity, provided appropriate body-related multisensory input.

A key advantage of our novel RLI paradigm is that it does not depend on referred sensations. Previous work has shown that the illusion is typically restricted to individuals with limb amputation who experience tactile referral from the residual to the phantom limb [16], a phenomenon occurring in roughly one sixth of cases and rarely involving precise point-to-point mappings [17]. Consequently, the only prior neuroimaging study on the RLI in individuals with limb amputation was limited to two individuals with upper limb amputation [18]. Our approach circumvents this constraint by always applying stimulation to the residual limb and the corresponding region of an artificial leg. This induces ownership of the stimulated artificial leg, which then extends to the unstimulated artificial foot. As a result, most participants could be induced to experience an altered sense of body integrity in the present study. Thus, in the *IntSync* condition of Experiment 1 (i.e., observing an intact artificial limb that is synchronously touched with the real residual limb), 79.4% of participants reported ownership of the stimulated artificial leg—similar to rates in non-amputated samples (e.g [6–9]). For the unstimulated artificial foot, the responder rate of 55.9% was lower, but similar (that is, 61.8%) to the responder rate in the *IntNonamp* condition of Experiment 2 (i.e., observing an intact artificial limb that is synchronously touched with the real non-amputated limb). This suggests comparable perceptual processes for both amputated and non-amputated limbs, challenging earlier claims of general sensory integration deficits for the residual limb [16]. This methodological strength also indicates new directions for prosthetic design. Whereas current strategies rely on eliciting referred sensations to enhance prosthesis embodiment, even with invasive procedures [32], our findings suggest that embodiment can be more easily supported by non-invasive sensory feedback systems that transmit tactile input applied to the socket as realistically as possible to the underlying residual limb skin. Such signals may induce ownership of the socket itself, potentially extending to the entire prosthesis. Our results suggest that the sense of body integrity could also be improved as a result.

Whole-brain fMRI analyses revealed activation in a widespread neural network involving bilateral PMv, SII+, MT+, and posterior parietal cortex. The pattern of results resembles previous findings on neural activity associated with the RLI. Thus, activity in PMv has previously been associated with the RLI and related paradigms, both in non-amputated participants (e.g. [6–8, 30, 33–36]), as well as two cases with upper limb amputation [18]. It has been shown that receptive fields in the PMv encode both the seen and the felt limb position [37]. This region is further involved in location encoding of tactile stimuli in a dynamic limb-centered reference frame [38], suggesting updating of own spatial body coordinates, based on incoming multisensory information [39, 40]. In previous RLI experiments, activity in this area has been shown to covary with illusory embodiment (e.g. [6–8]), and to contribute to the generation of a whole-body multisensory percept [24]. In the present study, however, the PMv was consistently activated and showed no clear variation as a function of experimental manipulation, despite clear perceptual effects. This suggests a rather unspecific involvement in the encoding of body-related multisensory stimulation in peripersonal space which may be rather indirectly linked to the sense of body integrity. Alternatively, this finding might reflect functional differences in premotor receptive fields for the arm and the leg. Different neural representations between upper and lower limbs were documented earlier. For instance, the primary receptive fields of the leg are larger and less densely distributed than those of the upper limbs [41, 42], especially in areas such as the thigh or the lower leg. This may reduce the accuracy of stimulus localization, which is, however, less critical for the function of the leg: while the hand is more strongly involved in multisensory processes (e.g., visual-haptic integration), leg functioning primarily relies on proprioceptive perception to coordinate body movements in space. Interestingly, a recent study on a foot version of the RLI in non-amputated individuals [43] also did not reveal illusion-associated PMv activity. Prospective studies should carefully examine the potentially distinct roles of the PMv in mediating the sense of ownership for the upper vs. lower limbs. Furthermore, it is important to consider that functional differences may exist in how the PMv is affected following upper vs. lower limb amputation, warranting a nuanced investigation into its differential alterations in these contexts.

Most importantly, this study identified a relationship between neural and perceptual interaction effects, indicating that sensory congruency and artificial limb appearance jointly support neuronal-perceptual coupling essential for body integrity processing. This relationship was specific for the left SPL, since we did not find this activity pattern for the other ROI analyses. Remarkably, the left SPL was found to be involved regardless of the side of amputation, suggesting distinct functional lateralization. The left SPL has been specifically related to body side-independent localization [44, 45] and awareness [46] of own body parts as well as the encoding of spatial relationships among body parts [47, 48]. Felician et al. [45] thus concluded that the left SPL processes the body’s spatial configuration in a flexible manner, providing crucial information to premotor cortex for action execution. Its involvement in the present experiment suggests that the RLI may dynamically engage multisensory processes specifically in response to conflicts with the physical (that is, impaired) body configuration. This interpretation carries important real-world implications for individuals with lower limb amputation using a prosthesis: if the left SPL can flexibly adapt to both an impaired (amputation) and an intact (prosthesis) body configuration, this adaptability could play a significant role in prosthesis-related motor planning. Prospective studies could thus investigate whether the left SPL represents a promising target for neurostimulation to enhance prosthesis embodiment and quality of prosthesis use. Additionally, future researchers could explore whether the behavioral deficits observed in individuals with limb amputation—specifically in processing spatial relationships among body parts [49]—are modulated by prosthesis use, thereby potentially emphasizing a dynamic role of the left SPL in response to specific interventions or experiences.

The fact that we observe comparably strong left-hemispheric SPL activity associated with the RLI induced at the residual limb or the non-amputated limb points to the involvement of general neural processes. Evidence from a lesion study in a patient with a left parietal stroke suggests that this region plays a crucial role in the transformation of intrinsic into extrinsic coordinate systems of limb localization [50]. The calibration of these coordinate systems is central to the RLI, as it requires resolving the conflict between intrinsic (proprioception, tactile perception of the own limb) and extrinsic sensory body representations (visual perception of the artificial limb) [51]. The found correlation between neural activity in the left SPL and perceptual consequences of the RLI suggests that integration capabilities of this brain region contribute to conscious body perception. Interestingly, a recent study on non-amputated individuals demonstrated that neural activity specifically within the left posterior parietal cortex conforms to a Bayesian function describing the likelihood that the brain attributes ownership to an artificial limb [35]. Notably, the coordinates identified in that study closely correspond to those reported in the present work. However, the present correlation between neural activity and RLI outcome was only medium in extent, suggesting that other processes, potentially more cognitive ones, may also contribute to perceived body integrity in individuals with lower limb amputation. Moreover, it remains an open question whether the left SPL activity observed in the present study actually reflects Bayesian causal inference of perceived body integrity, comparable to processes described by previous authors [35], and whether such processes contribute to real-life prosthesis experiences. Moreover, prospective studies must further investigate the extent to which multisensory integration processes for residual versus intact limbs are truly comparable. Our findings permit some informed speculation: although no FWE-corrected activity in the left SPL was observed for the main effect of artificial limb appearance (intact vs. impaired) in Experiment 2, a corresponding effect emerged in the extracted neural beta values (see Fig. 6B). This apparent discrepancy may result from differences in analytical approaches: brain imaging typically applies stringent voxel- and cluster-level significance thresholds, whereas extracted activity averages across multiple voxels under more liberal statistical criteria. Such divergent results may point to differences in focal activity between conditions, potentially reflecting amputation-related neuroplastic adaptations. Nevertheless, converging behavioral and neural evidence indicates that the processes are at least similar, and possibly identical. Conclusive resolution of this question will require specifically targeted future studies.

Future research should further clarify the potentially differential functions of the left and right SPL in conscious body perception. Evidence from individuals with body integrity dysphoria, specifically the xenomelia subtype, suggests a possible hemispheric specialization. Body integrity dysphoria is a rare neuropsychiatric condition, with the xenomelia subtype being characterized by an intense desire for amputation of a healthy limb that is often experienced as foreign or not belonging to oneself. Neuroimaging studies consistently highlight structural and functional alterations predominantly in the right SPL [20–23]. As we have identified the left SPL as crucial for artificial limb embodiment and the sense of body integrity in individuals with limb amputation, this may point to lateralized and fundamentally distinct neural mechanisms: the right SPL might be essential for maintaining a coherent own-body percept to prevent limb disembodiment, whereas the left SPL could facilitate adaptive neuroplastic processes that update and extend the body schema to incorporate artificial objects as part of the self. Understanding how these hemispheric differences arise and interact could provide crucial insights into the neural basis of normal and pathological body perception.

Several limitations must be considered. Previous results suggest that the RLI can be elicited in non-amputated and amputated individuals after an average of approximately 11 s of visuo-tactile stimulation [6, 18] and sustains for tens of seconds after visuo-tactile stimulation has ceased [52]. In the present study, we neither assessed the individual’s onset nor the offset of the RLI percept, which could have an influence on the fMRI results. However, meaningful activation patterns found here seem plausible, also in terms of the previous results for non-amputated participants based on a similar fMRI methodology [30]. Nevertheless, neglecting individual dynamics in perception may contribute to the present study’s subtle differences in key regions such as the PMv across conditions (e.g [6–8]). While both the PMv and the SPL are involved in multisensory processing, it is the posterior parietal cortex—particularly the SPL—that demonstrates heightened responsiveness specifically to visuo-tactile information [53]. Notably, this increased activity is largely confined to the visuo-tactile stimulation period and does not extend beyond it. This difference in the functioning of the SPL and the PMv may account for the absence of distinct effects in the latter region. Neglecting the on- and offsets of illusory perceptions may also account for the non-specific involvement of MT + and SII + observed in the present study. This likely reflects responses to the swiping movement of the visual brush [30] and to tactile events, with the latter being particularly influenced by enhanced spatial attention [54, 55] that was specifically promoted through our experimental instruction. However, investigating the onsets and offsets of illusory body experiences (e.g. [6, 43]) typically requires a pre-test of the RLI, during which participants are explicitly informed about the perceptual specifics of the illusion under study and instructed to continuously monitor these throughout the main experiment. Since top-down cognitive load has been shown to modulate embodiment experiences in the RLI [56], it is reasonable to assume that focusing attention on the on- and offset of the illusion could per se influence neural responses. Therefore, our approach may yield results that are relatively unbiased. However, the omission of pre-sampling also led to the inclusion of low responders, thereby reducing the mean perceptual responses. To allow comparison with previous studies, we introduced the category of “overall responders”, defined as participants who ranked in the upper half of the response spectrum across all perceptual measures. More than half of the participants (52.9%) fell into this category, which is still a higher proportion than reported in previous studies. Presenting the results of both groups in the present study may help readers in contextualizing and critically evaluating the findings.

The heterogeneity of the current sample regarding amputation side represents another interpretative challenge. While some previous studies used to laterally flip the brain images of individuals with right-sided or left-sided amputation, respectively, to harmonize all images in terms of amputation side (e.g., [57, 58]), empirical evidence regarding the lateralization of neurocognitive processes prohibits such an approach. In the present study, using lateral flipping would have led to the cancellation of left-sided SPL activity. Finally, the lack of a non-amputated control group limits the conclusiveness of results. Thus, it remains open how specific the reported results are for individuals with limb amputation. Results of Experiment 2 suggest that the multisensory and neural processes for a residual and a non-amputated limb are comparable. However, without a control group, an alternative interpretation is that adaptive brain changes after limb amputation may equally affect RLI responsiveness for both the residual and the non-amputated limb.

## Conclusion

The results of the present study suggest that a majority of individuals with lower limb amputation may be able to flexibly recalibrate their sense of body integrity in response to appropriate multimodal input, with multisensory integration in the left SPL contributing to the percept of bodily wholeness. These findings may advance our understanding of neurobehavioral dynamics following limb amputation and may help to explain and facilitate the embodiment of prosthetic limbs through multimodal sensory stimulation (e.g., [32, 59–61]). This is of clinical relevance, as greater prosthesis embodiment has been associated with several improved rehabilitative outcomes (e.g., [3, 4, 62]). The present findings may point to a straightforward way of enabling more individuals with amputation to benefit from the perception-dependent positive effects of prosthesis use through non-invasive sensory feedback. However, whether the induction of experimental embodiment generalizes to habitual prosthesis experiences, and whether the identified principles in individuals with lower limb amputation transfer to those with upper limb amputation, remain questions for future research.

## Supplementary Information

Supplementary Material 1.

## Data Availability

Data are available from the corresponding author upon reasonable request.
