# Supplementary material for: Feeling (in)complete: neural correlates of perceived body integrity in individuals with lower limb amputation
Source: J Neuroeng Rehabil. 2026 Jan 5;23:38. doi: 10.1186/s12984-025-01817-3 (PMC12849752; doi:10.1186/s12984-025-01817-3)
Supplement: Supplementary file 1 — Supplementary Material 1. [file 12984_2025_1817_MOESM1_ESM.docx]

**Feeling (in)complete: neural correlates of perceived body integrity in lower limb amputees**

Robin Bekrater-Bodmann, Michaela Ruttorf

**– Additional file 1 –**

**Supplementary Table S1:** **Demographic and clinical characteristics of the included participants.**

| ID # | Sex | Age [years] | Side of amputation | Level of amputation | Reason of amputation | Time since amputation [years] | Excluded from fMRI analysis | Other medical conditions  (mostly drug-treated) | OR |
| --- | --- | --- | --- | --- | --- | --- | --- | --- | --- |
| 1 | f | 32 | R | transfemoral | cancer | 25 |  |  |  |
| 2 | f | 38 | L | transfemoral | cancer | 27 |  |  |  |
| 3 | m | 54 | L | transfemoral | injury | 25 |  | diabetes |  |
| 4 | m | 57 | R | transtibial | injury | 32 |  |  |  |
| 5 | m | 62 | L | transfemoral | injury, infection | 43 |  | arthritis, hypothyroidism, hypertension |  |
| 6 | m | 52 | R | transfemoral | cancer | 32 |  |  | × |
| 7 | m | 61 | L | transfemoral | injury | 20 |  |  | × |
| 8 | m | 60 | L | transtibial | injury | 44 |  | hypothyroidism |  |
| 9 | m | 55 | R | transtibial | injury | 42 |  | hypothyroidism | × |
| 10 | m | 67 | L | transfemoral | injury, infection | 51 |  |  | × |
| 11 | m | 58 | L | transtibial | injury | 17 |  |  | × |
| 12 | m | 53 | R | transtibial | injury | 51 | × |  |  |
| 13 | f | 50 | R | transfemoral | cancer | 26 |  | migraine | × |
| 14 | m | 57 | R | transtibial | injury | 25 |  |  |  |
| 15 | m | 63 | L | transfemoral | injury | 46 |  |  | × |
| 16 | m | 40 | L | transtibial | injury | 16 |  |  |  |
| 17 | f | 47 | R | transtibial | injury, infection | 24 |  |  | × |
| 18 | m | 43 | L | transfemoral | injury | --- |  | --- | × |
| 19 | m | 71 | L | transfemoral | injury | 41 |  | hypertension | × |
| 20 | m | 40 | L | transfemoral | cancer | 25 |  |  |  |
| 21 | m | 62 | R | transtibial | injury | 44 |  | atrial fibrillation | × |
| 22 | m | 62 | L | transtibial | injury | 45 |  |  | × |
| 23 | m | 71 | R | transfemoral | injury | 54 |  | hypertension |  |
| 24 | m | 61 | L | transtibial | infection | 54 |  | hypertension |  |
| 25 | m | 61 | L | transtibial | injury | 43 |  | diabetes | × |
| 26 | m | 56 | L | transfemoral | injury | 35 |  | hypertension |  |
| 27 | m | 54 | L | transtibial | injury | 35 |  |  | × |
| 28 | f | 61 | L | transfemoral | injury | 32 |  | migraine, hypothyroidism |  |
| 29 | f | 68 | L | transtibial | injury | 13 | × | diabetes | × |
| 30 | m | 49 | L | transfemoral | injury | 41 |  |  | × |
| 31 | m | 53 | L | transfemoral | injury | 36 |  |  |  |
| 32 | m | 65 | L | transfemoral | infection | 11 |  |  |  |
| 33 | m | 59 | R | transfemoral | infection | 10 |  | hypertension | × |
| 34 | f | 57 | R | transtibial | infection | 2 | × |  | × |

f = female; m = male; R = right; L = left; fMRI = functional magnetic resonance imaging; --- = missing information; OR = overall responder (for operationalization, see the main text)

**Analysis of touch referral data**

*Experiment 1*

A main effect of the factor *visuo-tactile stimulation*, *F*(1,33) = 67.42, *P* < 0.001, *η*^2^ = 0.67, with synchronous stimulation being associated with significantly higher ratings of touch referral than asynchronous stimulation, proved the experimental variation of visuo-tactile synchrony working. The factor *artificial limb* showed no main effect, *F*(1,33) = 0.52, *P* = 0.47, *η*^2^ = 0.02, and there was no significant *artificial limb × visuo-tactile stimulation* interaction, *F*(1,33) = 0.01, *P* = 0.91, *η*^2^ < 0.001, suggesting that the touch was perceived similarly, independent of whether it was applied to an intact or an impaired artificial limb (see Suppl. Fig. S1A).

*
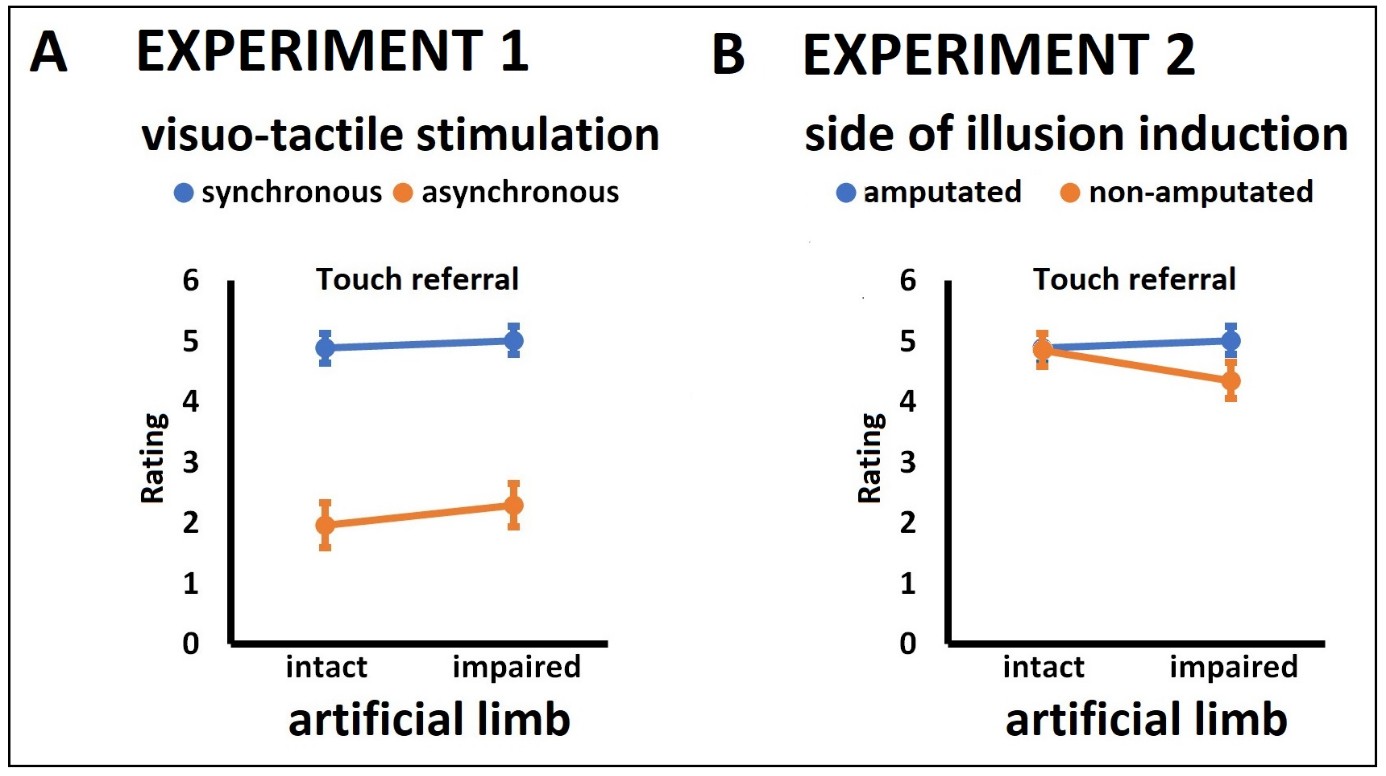
*

**Supplementary Figure S1:** Touch referral data in obtained from (**A**) Experiment 1 and (**B**) Experiment 2. Given are the mean ratings +/- standard error of the mean (higher values indicate higher agreement to items asking for the given measure). Displayed are non-transformed data.

*Experiment 2*

There was no main effect of the factor *artificial limb*, *F*(1,33) = 3.37, *P* = 0.08, *η*^2^ = 0.09. However, there was a main effect for the factor *body side*, *F*(1,33) = 6.04, *P* = 0.02, *η*^2^ = 0.15. Further, there was a significant *artificial limb × body side* interaction, *F*(1,33) = 4.96, *P* = 0.03, *η*^2^ = 0.13. The interaction was driven by significantly reduced touch referral in the *ImpNonamp* condition compared to both conditions implemented on the amputated side, both *T*(33) ≥ -2.92, both *P_Bonf_* ≤ 0.01, both *d* ≥ 0.50, while there was no significant difference to the *IntNonamp* condition, *T*(33) ≥ -2.43, *P_Bonf_* = 0.06, *d* ≥ 0.42, suggesting that differences in the perception of visuo-tactile stimulation mainly occurred under the unusual circumstance of observing an impaired limb on the non-amputated body side. These results are visualized in Suppl. Fig. S1B.

**Supplementary Table S2: Results for whole-brain analysis – contrast *IntAsync*.**

| Functional unit | Atlas Label (AAL) | Hemi-sphere | MNI coordinates  [mm]  [mm] | | | Peak *t*-value | Voxels per Cluster |
| --- | --- | --- | --- | --- | --- | --- | --- |
|  |  |  | x | y | z |  |  |
|  | Postcentral | R | 55 | -20 | 35 | 7.43 | 472 |
| SII+ | Supra Marginal | R | 60 | -27 | 19 | 10.92 |  |
|  |  | R | 48 | -32 | 24 | 8.12 |  |
|  |  | L | -58 | -20 | 35 | 9.26 | 310 |
|  | Postcentral | L | -58 | -18 | 21 | 8.79 |  |
|  | Parietal Inf | L | -42 | -45 | 56 | 6.23 | 25 |
| MT+ | Temporal Mid | R | 48 | -61 | 3 | 8.75 | 160 |
|  |  | L | -53 | -68 | 10 | 7.78 | 303 |
|  |  | L | -44 | -66 | 5 | 7.30 |  |
|  |  | L | -58 | -52 | 5 | 6.98 |  |
|  | Occipital Mid | L | -44 | -71 | -2 | 8.72 |  |
|  | Frontal Mid 2 | R | 39 | -2 | 60 | 6.96 | 30 |
|  |  | L | -42 | 8 | 51 | 5.93 | 257 |
| PMv | Precentral | L | -42 | -6 | 56 | 8.74 |  |
|  |  | L | -53 | 3 | 40 | 7.17 |  |
|  |  | L | -58 | 8 | 28 | 6.46 |  |
|  |  | R | 55 | 8 | 42 | 6.63 | 92 |
|  | Frontal Inf Tri | R | 30 | 26 | 30 | 6.47 |  |
|  | Temporal Sup | L | -58 | -38 | 21 | 6.98 | 47 |
|  | Supp Motor Area | R | 2 | 1 | 63 | 6.73 | 39 |

All peak t-values listed are significant after whole-brain correction for multiple comparisons using a family-wise error rate (*P* < 0.05, FWE-corrected at voxel level) with extent threshold *k* > 20 voxels. For clusters *k* > 100, all significant peak values are denoted. Functional unit refers to the brain regions (MT+, medial temporal complex; PMv, ventral premotor cortex; SII+, secondary somatosensory cortex and its vicinity, including Rolandic operculum, supramarginal cortex, and adjacent parts of the inferior parietal lobule) that were consistently identified and is intended to facilitate the assignment to description in the results section. Note that the analyses did not reveal significant activity in the superior parietal lobule. L/R denotes the left and right hemisphere, respectively. Atlas labels according to Automatic Anatomical Labelling (AAL) Atlas 3. MNI, Montreal Neurological Institute.

**Supplementary Table S3: Results for whole-brain analysis – contrast *ImpSync / ImpAmp*.**

| Functional unit | Atlas Label (AAL) | Hemi-sphere | MNI-Coordinates | | | Peak *t*-value | Voxels per Cluster |
| --- | --- | --- | --- | --- | --- | --- | --- |
|  |  |  | x | y | z |  |  |
|  | Postcentral | R | 60 | -15 | 33 | 6.03 | 207 |
| SII+ | Rolandic Oper | R | 55 | -29 | 19 | 7.99 |  |
|  | Supra Marginal | R | 62 | -20 | 24 | 7.25 |  |
|  | Supra Marginal | L | -60 | -20 | 42 | 9.44 | 294 |
|  | Postcentral | L | -60 | -18 | 24 | 8.52 |  |
|  | Parietal Inf | L | -39 | -48 | 58 | 6.49 | 47 |
| MT+ | Temporal Mid | R | 46 | -61 | 3 | 9.77 | 265 |
|  |  | L | -51 | -71 | 1 | 9.25 | 268 |
|  |  | L | -44 | -66 | 5 | 7.15 |  |
|  | Occipital Mid | L | -30 | -91 | -6 | 7.86 | 37 |
|  | Occipital Inf | R | 32 | -89 | -4 | 8.02 | 49 |
| PMv | Precentral | L | -53 | 5 | 42 | 7.74 | 43 |
|  |  | L | -42 | -9 | 60 | 7.46 | 24 |
|  |  | R | 57 | 10 | 37 | 7.19 | 152 |
|  | Frontal Mid 2 | R | 39 | -2 | 63 | 7.09 |  |
|  |  | R | 41 | 3 | 56 | 6.11 |  |
|  |  | R | 48 | 3 | 51 | 5.83 |  |

All peak *t*-values listed are significant after whole-brain correction for multiple comparisons using a family-wise error rate (*P* < 0.05, FWE-corrected at voxel level) with extent threshold *k* > 20 voxels. For clusters *k* > 100, all significant peak values are denoted. Functional unit refers to the brain regions (MT+, medial temporal complex; PMv, ventral premotor cortex; SII+, secondary somatosensory cortex and its vicinity, including Rolandic operculum, supramarginal cortex, and adjacent parts of the inferior parietal lobule) that were consistently identified and is intended to facilitate the assignment to description in the results section. Note that the analyses did not reveal significant activity in the superior parietal lobule. L/R denotes the left and right hemisphere, respectively. Atlas labels according to Automatic Anatomical Labelling (AAL) Atlas 3. MNI, Montreal Neurological Institute.

**Supplementary Table S4: Results for whole-brain analysis – contrast *ImpAsync*.**

| Functional unit | Atlas Label (AAL) | Hemi-sphere | MNI coordinates | | | Peak *t*-value | Voxels per Cluster |
| --- | --- | --- | --- | --- | --- | --- | --- |
|  |  |  | x | y | z |  |  |
|  | Parietal Inf | L | -39 | -50 | 58 | 7.33 | 90 |
|  |  | L | -39 | -59 | 56 | 6.59 |  |
| SII+ | Supra Marginal | R | 62 | -27 | 19 | 10.06 | 296 |
|  |  | L | -58 | -41 | 24 | 8.36 | 463 |
|  |  | L | -58 | -45 | 30 | 6.99 |  |
|  | Parietal Inf | L | -53 | -22 | 40 | 8.09 |  |
|  | Temporal Sup | L | -53 | -32 | 17 | 7.20 |  |
|  | Postcentral | L | -60 | -18 | 26 | 9.06 |  |
|  |  | R | 18 | -43 | 70 | 6.59 | 38 |
| MT+ | Temporal Mid | R | 48 | -61 | 3 | 8.82 | 137 |
|  |  | L | -42 | -66 | 5 | 8.57 | 177 |
|  |  | L | -53 | -63 | 7 | 8.43 |  |
|  | Occipital Mid | L | -46 | -73 | -2 | 6.82 |  |
| PMv | Precentral | R | 53 | 8 | 47 | 7.54 | 70 |
|  |  | L | -53 | 5 | 42 | 7.03 | 81 |
|  |  | L | -39 | -6 | 54 | 6.62 | 42 |
|  | Frontal Mid 2 | R | 39 | -2 | 63 | 7.22 | 22 |
|  | Cerebellum Crus1 | R | 14 | -82 | -22 | 6.72 | 27 |

All peak *t*-values listed are significant after whole-brain correction for multiple comparisons using a family-wise error rate (*P* < 0.05, FWE-corrected at voxel level) with extent threshold *k* > 20 voxels. For clusters *k* > 100, all significant peak values are denoted. Functional unit refers to the brain regions (MT+, medial temporal complex; PMv, ventral premotor cortex; SII+, secondary somatosensory cortex and its vicinity, including Rolandic operculum, supramarginal cortex, and adjacent parts of the inferior parietal lobule) that were consistently identified and is intended to facilitate the assignment to description in the results section. Note that the analyses did not reveal significant activity in the superior parietal lobule. L/R denotes the left and right hemisphere, respectively. Atlas labels according to Automatic Anatomical Labelling (AAL) Atlas 3. MNI, Montreal Neurological Institute.

**Supplementary Table S5: Results for whole-brain analysis – contrast *IntNonamp*.**

| Functional unit | Atlas Label (AAL) | Hemi-sphere | MNI coordinates | | | Peak *t*-value | Voxels per Cluster |
| --- | --- | --- | --- | --- | --- | --- | --- |
|  |  |  | x | y | z |  |  |
| SPL | Parietal Sup | L | -21 | -45 | 74 | 7.04 | 25 |
|  |  | R | 16 | -68 | 60 | 6.17 | 32 |
|  |  | R | 39 | -55 | 56 | 7.43 | 222 |
|  | Parietal Inf | R | 32 | -50 | 51 | 6.78 |  |
|  |  | L | -39 | -48 | 60 | 8.23 | 427 |
|  |  | L | -28 | -57 | 53 | 7.69 |  |
|  |  | L | -37 | -61 | 53 | 6.65 |  |
|  |  | L | -25 | -48 | 49 | 6.10 |  |
|  |  | L | -48 | -52 | 49 | 5.72 |  |
|  | Temporal Sup | L | -46 | -29 | 19 | 7.12 | 313 |
|  | Parietal Inf | L | -53 | -22 | 21 | 8.66 |  |
| SII+ |  | L | -58 | -27 | 47 | 6.91 |  |
|  | Supra Marginal | L | -55 | -22 | 21 | 8.27 |  |
|  |  | L | -51 | -38 | 26 | 5.68 |  |
|  | Rolandic Oper | R | 57 | -29 | 19 | 6.76 | 28 |
| MT+ | Occipital Mid | L | -48 | -71 | -2 | 9.27 | 248 |
|  | Temporal Mid | R | 46 | -61 | 3 | 7.73 | 173 |
|  | Temporal Inf | R | 50 | -66 | -6 | 7.59 |  |
| PMv | Precentral | R | 57 | 8 | 37 | 6.59 | 75 |
|  |  | L | -55 | 3 | 40 | 7.22 | 31 |
|  |  | L | -32 | 9 | 56 | 6.46 | 28 |

All peak *t*-values listed are significant after whole-brain correction for multiple comparisons using a family-wise error rate (*P* < 0.05, FWE-corrected at voxel level) with extent threshold *k* > 20 voxels. For clusters *k* > 100, all significant peak values are denoted. Functional unit refers to the brain regions (MT+, medial temporal complex; PMv, ventral premotor cortex; SII+, secondary somatosensory cortex and its vicinity, including Rolandic operculum, supramarginal cortex, and adjacent parts of the inferior parietal lobule) that were consistently identified and is intended to facilitate the assignment to description in the results section. Note that the analyses also reveal significant activity in the superior parietal lobule (SPL). L/R denotes the left and right hemisphere, respectively. Atlas labels according to Automatic Anatomical Labelling (AAL) Atlas 3. MNI, Montreal Neurological Institute.

**Supplementary Table S5: Results for whole-brain analysis – contrast *ImpNonamp*.**

| Functional unit | Atlas Label (AAL) | Hemi-sphere | MNI coordinates | | | Peak *t*-value | Voxels per Cluster |
| --- | --- | --- | --- | --- | --- | --- | --- |
|  |  |  | x | y | z |  |  |
|  | Parietal Inf | R | 37 | -48 | 47 | 6.24 | 198 |
|  |  | R | 44 | -45 | 51 | 6.18 |  |
| SII+ |  | R | 41 | -52 | 40 | 5.47 |  |
|  | Supra Marginal | R | 46 | -36 | 42 | 5.76 |  |
| SPL | Parietal Sup | R | 34 | -57 | 58 | 6.93 |  |
|  |  | L | -30 | -59 | 60 | 6.32 | 699 |
| SII+ | Supra Marginal | L | -58 | -20 | 40 | 9.11 |  |
|  |  | L | -58 | -22 | 24 | 6.42 |  |
|  | Parietal Inf | L | -39 | -48 | 58 | 8.91 |  |
|  |  | L | -42 | -48 | 47 | 6.70 |  |
|  |  | L | -48 | -43 | 49 | 6.11 |  |
| MT+ | Temporal Mid | L | -51 | -71 | 1 | 9.44 | 220 |
|  |  | R | 46 | -64 | 3 | 7.52 | 179 |
|  | Temporal Inf | R | 50 | -64 | -6 | 6.99 |  |
| PMv | Precentral | R | 55 | 8 | 37 | 6.51 | 58 |
|  |  | L | -53 | 3 | 37 | 10.06 | 149 |
|  |  | L | -32 | -2 | 60 | 6.03 | 54 |
|  | Frontal Mid 2 | R | 44 | 31 | 35 | 5.82 | 27 |
|  | Temporal Sup | R | 55 | -32 | 19 | 8.31 | 99 |

All peak *t*-values listed are significant after whole-brain correction for multiple comparisons using a family-wise error rate (*P* < 0.05, FWE-corrected at voxel level) with extent threshold *k* > 20 voxels. For clusters *k* > 100, all significant peak values are denoted. Functional unit refers to the brain regions (MT+, medial temporal complex; PMv, ventral premotor cortex; SII+, secondary somatosensory cortex and its vicinity, including Rolandic operculum, supramarginal cortex, and adjacent parts of the inferior parietal lobule) that were consistently identified and is intended to facilitate the assignment to description in the results section. Note that the analyses also reveal significant activity in the superior parietal lobule (SPL). L/R denotes the left and right hemisphere, respectively. Atlas labels according to Automatic Anatomical Labelling (AAL) Atlas 3. MNI, Montreal Neurological Institute.
